# Gene therapy as an innovative approach to the treatment of hemophilia B—a review

**DOI:** 10.1007/s13353-025-00952-w

**Published:** 2025-04-03

**Authors:** Kinga Wróblewska, Dominika Bieszczad, Magdalena Popławska, Karolina Joanna Ziętara, Monika Zajączkowska, Agata Filip

**Affiliations:** 1 Students’ Scientific Association at the Department of Cancer Genetics with Cytogenetics Laboratory, Medical University of Lublin, Ul. Radziwiłłowska 11, 20-080 Lublin, Poland; 2 Student Scientific Association at the Department of Psychology, Faculty of Medicine, Medical University of Lublin, 20-093 Lublin, Poland; 3 Department of Cancer Genetics, Cytogenetics Laboratory, Medical University of Lublin, 20-080 Lublin, Poland

**Keywords:** Hemophilia B, Gene therapy, Viral vectors, Etranacogene dezaparvovec

## Abstract

Hemophilia B is a disease that affects the human coagulation system, causing the absence or deficiency of coagulation factor IX, which may manifest itself in uncontrolled bleeding that is life-threatening to patients. Due to its inheritance, the disease more often affects men, and the severity of symptoms directly correlates with the concentration of the missing factor IX; hence, the aim of therapy is to maintain it at a level that allows for sufficient hemostasis. The basic model of treatment offered to patients is based on primary prevention with coagulation factor IX with a prolonged half-life, which, however, does not solve the numerous problems faced by patients. An innovative proposal that, despite initial concerns, is becoming more and more popular every day is the recently approved genetic therapy in Europe, which uses viral vectors to transfer the correct gene that encodes coagulation factor IX. The introduction of a recombinant gene in place of its defective counterpart seems to be a promising solution and the beginning of a new era in which genetic therapies have a chance to develop their full potential and replace existing therapeutic regimens.

## Introduction

Hemophilia is a group of rare congenital disorders caused by the absence or deficiency of specific blood clotting factors produced in the liver. There is a distinction between hemophilia A, B, or C, in which there are mutations in the genes encoding Factor VIII (FVIII), Factor IX (FIX), or Factor XI (FXI), respectively. The prevalence of hemofilia A is reported as 1 in 5000 in the male population and 1 in 10,000 overall. The prevalence of hemofilia B is commonly reported as one in 30,000 males. Hemofilia C is much less common than the other two types. Based on the data collected by World Federation of Haemophilia in 2021, these disorders affect an estimated 815,100 people worldwide with approximately 27,690 individuals experiencing severe forms of the disease (Berntorp et al. 2021a; World Federation of Hemophilia 2020; Stonebraker et al. 2010). The genes encoding FVIII (*F8*) and FIX (*F9*) are located on the long arm of the X chromosome (Xq28 and Xq27.1, respectively) and are inherited in an X-linked recessive manner. The *FXI* gene is located on the long arm of chromosome 4 (4q35.2) and most cases of severe deficiency seem to follow an autosomal-recessive inheritance pattern (Lewandowska and Connors 2021; Kravtsov et al. 2004). The chromosomal location and mode of inheritance influence the manifestation of the disease in both sexes. Only individuals with a full expression of the mutated gene, namely, hemizygous men and homozygous women with an abnormal *F8* or *F9*, exhibit hemophilia. Heterozygous women, who possess one wild-type allele on their second X chromosome that can compensate for the deficiency, are typically considered carriers of the defective genes (Miller and Bean 2021; d’Oiron et al. 2021).

The severity of the symptoms directly correlates with the level of clotting factor in the blood serum. Based on this, hemophilia can be classified as severe (< 1 units/deciliter (µ/dL)), moderate (1–5 µ/L), or mild (> 5 and < 40 µ/dL). For patients affected by hemophilia A, about 40% of patients have a severe form of the disease, about 10% have a moderate form, and the remaining 50% have a mild form of hemophilia. In the case of hemophilia B, this distribution is not so well established (Berntorp et al. 2021a). Severe phenotype is characterized by uncontrolled, life-threatening bleeding (such as intracranial, perioperative, and post-traumatic bleeding) and recurrent, spontaneous bleeding into muscles and joints, potentially leading to arthropathy. In contrast, patients with moderate and mild phenotype are less prone to spontaneous bleeding episodes but remain at risk of bleeding following injury (Nathwani 2022). Additionally, women with hemophilia may experience symptoms such as excessive menstrual and perinatal bleeding and an increased risk of miscarriages (Miller and Bean 2021; d’Oiron et al. 2021).

The current standard of care for patients with hemophilia includes regular primary prophylaxis with recombinant or plasma-derived factors VIII or IX, aiming to maintain their levels above 1% of normal activity (Aledort et al. 2019). It is crucial that this prevention is maintained throughout patients’ lives, starting from early childhood, to minimize the potential risk of health complications, particularly the development of arthropathy (Nathwani 2022). Although prophylaxis is the preferred therapeutic option, administered clotting factors have a relatively short half-life, providing only temporary correction of hemostasis and requiring frequent administration. This issue has been addressed by developing molecules with an extended half-life, now used in current therapeutic regimens. This advancement was achieved through conjugation with polyethylene glycol and fusion with proteins such as albumin or the Fc fragment of the IgG1 antibody (Aledort et al. 2019; Ozelo and Yamaguti-Hayakawa 2022; Hart et al. 2022; Kaczmarek et al. 2024). Despite the clear benefits of treatment with clotting factors, patients undergoing this therapy may experience side effects, with one of the most significant being the development of polyclonal alloantibodies, primarily of the IgG class (also known as inhibitors), which neutralize the activity of FVIII or FIX. About 20–30% of patients with severe phenotype and about 5–10% of patients with mild to moderate hemophilia A develop inhibitors, whereas in hemophilia B, alloantibodies occur in only 5% of patients with its severe form (Berntorp et al. 2021b; Ljung et al. 2019; Carcao et al. 2019).

The presence of inhibitors, particularly those with high titers above 5 BU/ml, is associated with an increased risk of bleeding, severe arthropathy leading to disability, and higher risk of mortality. Inhibitor eradication can be achieved through immune tolerance induction (ITI), which involves regular administration of high doses of clotting factor VIII/IX concentrates (Carcao et al. 2019). Additionally, in patients who develop inhibitors and suffer from acute bleedings, alloantibody “bypassing agents” such as activated prothrombin complex concentrate (aPCC) or recombinant activated factor VII (rFVIIa) can be administered. In the case of patients with hemophilia A and inhibitors, particularly helpful is the bispecific antibody emicizumab, which acts as a link between activated factor IX and factor X to restore the activity of the missing activated factor VIII. Emicizumab does not induce or enhance the formation of direct factor VIII inhibitors and is more effective than the aforementioned bypassing agents, although it is not used in hemophilia B (Carcao et al. 2019; Meeks and Leissinger 2019; Hoffmann-La Roche Limited 2023). ITI is less effective in patients with hemophilia B and inhibitors, and these patients are also about 10 times more likely to experience side effects such as allergic reactions and nephrotic syndrome. Therefore, ITI trials in hemophilia B patients with inhibitors should be conducted with caution (Ljung et al. 2019).

Hemophilia is an example of a disease where gene therapy may offer an effective treatment method. Its pathogenesis is based on a single gene defect, leading to the absence of a specific protein. Thus, minimal expression of the gene for FVIII or FIX, resulting in the synthesis of the appropriate proteins and a slight increase in coagulation factors in the blood (up to 5%), can significantly alter the course of the disease (Nathwani 2022; Leebeek and Miesbach 2021). Gene therapy represents a new and potentially effective method of treating hemophilia, potentially avoiding the complications associated with other therapies (Okaygoun et al. 2021; Muczynski and Nathwani 2024).

## Historical view

**Fig. 1:**
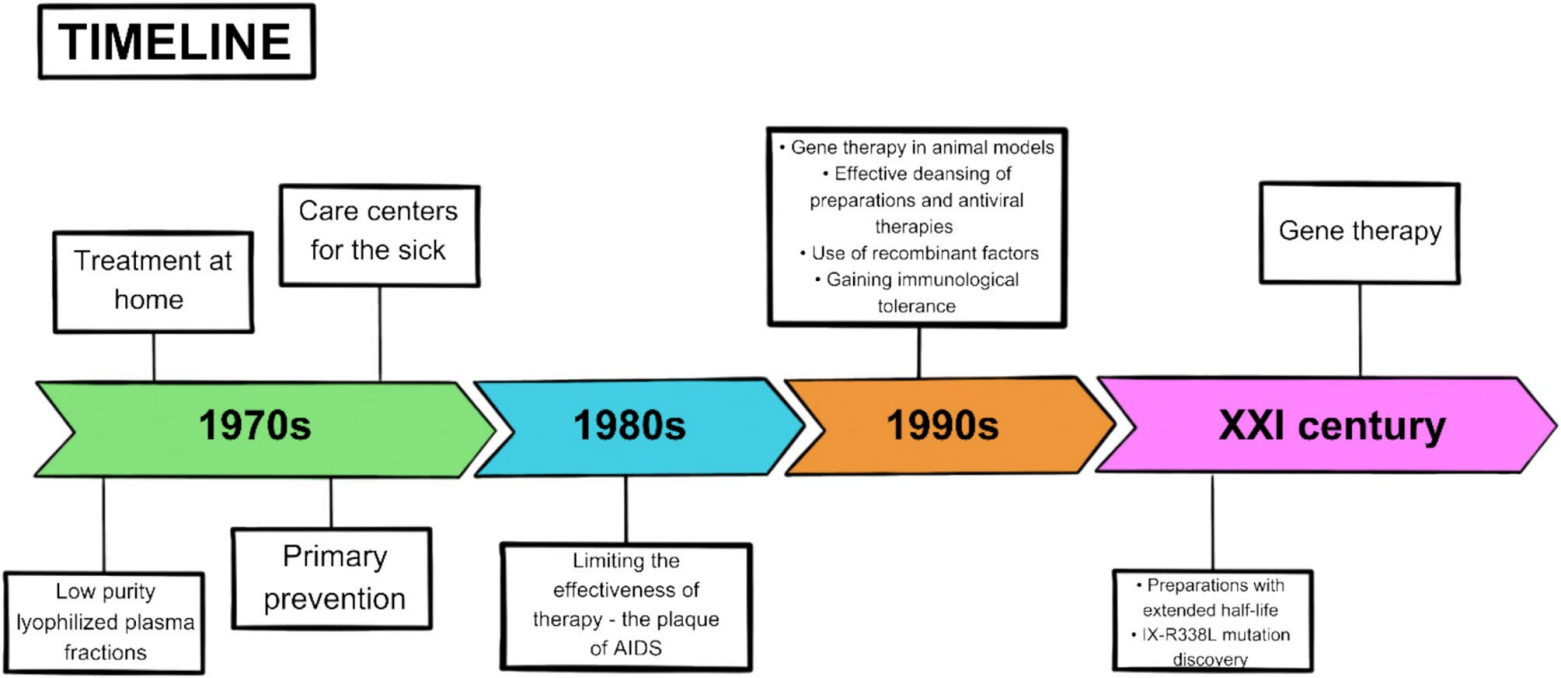
A timeline of milestones in the development of hemophilia B treatment

The treatment of patients with hemophilia has undergone enormous changes over the decades. The first attempts to create a model of therapy for this disease took place in 1964, when large amounts of FVIII were detected in the cryoprecipitate fraction from plasma. Therapies developed at that time were based on this discovery, involving the administration of cryoprecipitate enriched with FIX for hemophilia B patients and early concentrates. However, these therapies did not significantly reduce the mortality rate of hemophilia patients or extend their lives substantially, necessitating further research and development of new methods. The primary issue reducing the effectiveness of these preparations was their significant contamination, leading to immune responses in patients who received the infusion. The 1970s marked an evolution in therapy: the volume of fluid used was reduced, infusions were replaced by injections, and self-administration of the preparation at home became possible. Regular use of FIX began, initiating the era of prophylaxis for hemophilia symptoms. These changes provided hope for a better quality of life, yet also introduced risks such as thromboembolic events and contamination with pathogens, including hepatitis C virus and HIV. Overall, therapies up to the point of effective plasma purification and the use of advanced diagnostic and therapeutic methods brought minimal long-term benefits to only a few patients. Progress since the 1990s has been spectacular and has allowed hemophilia treatment to truly flourish. Gene therapy, in particular, represents a significant breakthrough. It is the only therapeutic option that offers hope for a cure by addressing the root cause, specifically administering a factor IX transgene. This approach holds the promise of a better life for many patients suffering from genetically determined diseases with a single gene defect (Mannucci 2008, 1993; Franchini and Mannucci 2022, 2014; Kasper 2005). The most important achievements in the treatment of hemophilia is shown in Fig. 1.

## Early gene therapy attempts

Gene therapy for hemophilia began in the 1990s, with several vectors tested to introduce the FIX transgene into cells, including retroviral, adenoviral, adeno-associated virus (AAV), and plasmid DNA vectors. Early attempts involved transducing the liver, skeletal muscles, and skin fibroblasts with vectors (Manno et al. 2006; Herzog et al. 1997). The initial stages of gene therapy development were challenging. Early animal trials involved the use of retroviruses, which were ineffective as they could not infect post-mitotic cells. Similarly, adenoviruses, despite their ability to integrate into the host genome, proved ineffective due to the immune system’s reactive response. This response led to the elimination of cells infected with adenoviruses containing the transgene, resulting in only transient therapeutic effectiveness (Dai et al. 1995; Walter et al. 1996). Adeno-associated virus (AAV) also presented immunogenicity issues, though less severe. Studies showed that AAV-2 had the highest immunogenicity, with 59% seroprevalence of neutralizing antibodies, AAV-5 had the lowest seroprevalence at 3.2%. This finding led researchers to focus on AAV-5 for gene therapy, administering it intravenously (Manno et al. 2006; Boutin et al. 2010). Various studies and alternative approaches, such as expressing FIX in platelets, were explored but ultimately not pursued for further development (Zhang et al. 2007).

## Development of AAV therapy

From a genetic standpoint, adeno-associated virus therapy (AAV) involves introducing a functional protein gene, or transgene, into the patient’s somatic cells using a carrier molecule, i.e., a vector. The vector comprises a capsid and an expression cassette containing, among others, the transgene and promoters (Au et al. 2022). AAV vectors are derived from a single-stranded non-pathogenic DNA virus from the Parvoviridae family. Wild-type adenovirus (wtAAV) has a high safety profile, is considered non-pathogenic for humans, and poorly immunogenic, requiring a helper virus (e.g., herpes virus) to replicate in the host body (Nathwani 2022; Samelson-Jones and George 2023). The AAV virus genome consists of two open reading frames, *rep* and *cap*, flanked by inverted terminal repeat sequences (ITR). *Rep* encodes proteins responsible for virus replication, and *cap* encodes structural proteins that form the capsid (Sabatino et al. 2022). Over 100 naturally occurring AAV serotypes are known, defined by capsid-building proteins responsible for cellular tropism and the host’s immune response. Both naturally occurring serotypes and bioengineered capsids are being evaluated for gene therapy for hemophilia (Pei et al. 2010).

AAV5 is most frequently used in clinical trials and is considered the most universal of all serotypes due to its low risk of recognition and neutralization by antibodies (NAB). The seroprevalence of anti-AAV NAB in the population is estimated to be between 3 and 40%. Additionally, it was shown that in hemophilia B patients with circulating anti-AAV5 antibodies, factor IX transduction was effective and comparable, regardless of the level of pre-existing anti-AAV5 NAB levels (Majowicz et al. 2019). Genetic treatment of hemophilia uses recombinant adenovirus (rAAV)-based vectors lacking wild-type viral coding sequences to lower the potential for triggering a cellular immune response to foreign viral proteins (Nathwani 2022; Pei et al. 2010). The rep and cap sequences are replaced by a single-stranded transgene expression cassette containing the therapeutic gene (factor VIII or factor IX), and transcription regulatory elements, such as a promoter sequence, to increase transgene expression in specific types of cells, particularly hepatocytes. The expression cassette is flanked by palindromic inverted terminal repeats, enabling transcription. After binding to the hepatocyte, endocytosis leads to the vector’s internalization with the host cell. The capsid is degraded, and the transgene is released, forming episomal DNA in the host cell thanks to terminal repeat (ITR) sequences. Episomes enable stable, long-time expression of the transgene in non-dividing cells without the damaging the target cell. The complementary strand to the vector’s single-stranded DNA is synthesized, resulting in the production of functional coagulation factor VIII or IX (Sidonio et al. 2021). Importantly, stable expression of factor IX protein has been shown to persists even for over 11 years after a single administration of the scAAV8-co F9 vector in patients with hemophilia B (George et al. 2020).

**Fig. 2:**
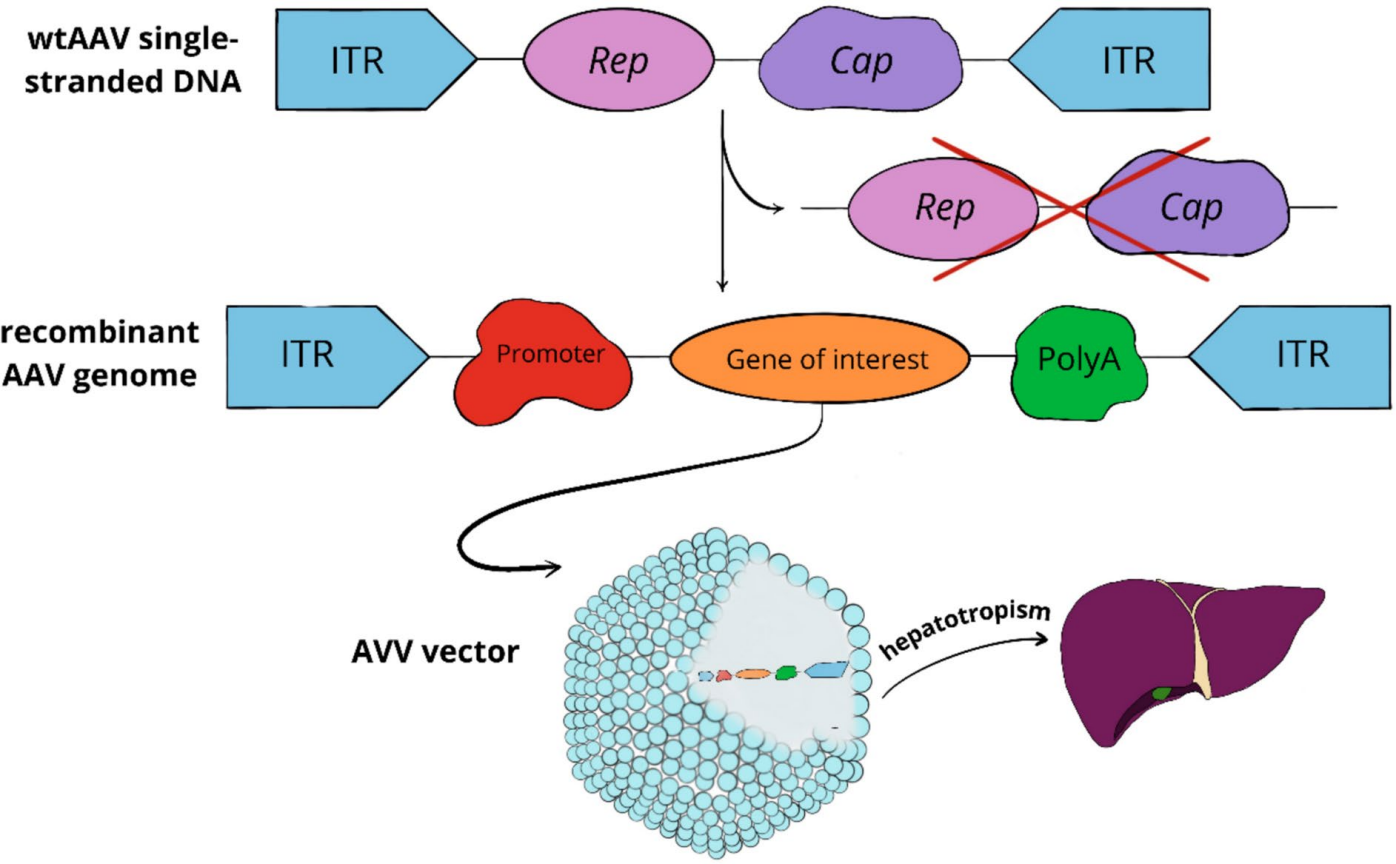
Naturally occurring adenovirus consists of two open reading frames—rep and cap, flanked by ITR sequences. The rep and cap sequences are removed from the wtAAV virus DNA and replaced with a transgene consisting of the gene of interest—gene of factor IX, other transcription regulatory sequences—polyA and promoter. Eventually the AAV vector consists of a recombinant AAV genome and capsid, showing tropism to the hepatocytes

The first drug utilizing the potential of genetic therapy for hemophilia B is etranacogene dezaparvovec, which contains a recombinant AAV serotype 5 vector targeted at liver cells and a codon-optimized variant of the human factor IX gene (Padua) (Anguela and High 2024). Phase III studies confirmed the therapy’s effectiveness showing persistent endogenous expression of factor IX for at least 18 months after a single intravenous infusion (Heo 2023; Sekayan et al. 2023). Subsequent reports indicate a 64% reduction in the annual bleeding rate in patients up to 2 years after the infusion, with no need to use prophylaxis with factor IX-containing preparations (Castaman et al. 2023). Long-term analysis predicts factor IX expression for up to 25 years after administration of a single full dose, promising a bright future for hemophilia patients (Shah et al. 2023). Figure 2 shows the general structure of the AAV therapy vector.

## Risk of hepatocellular carcinoma (HCC)

Hepatocellular carcinoma (HCC) is a significant cause of increased mortality in the elderly population with hemophilia. Major risk factors for HCC include chronic alcohol consumption, diabetes, non-alcoholic steatohepatitis, and hepatitis B virus (HBV) and hepatitis C virus (HCV) infection. Approximately 80% of HCC cases are mainly associated with chronic infection with HBV or HCV. Before the introduction of virally inactivated coagulation factors, most hemophiliacs developed chronic HCV infection (Shetty et al. 2016; Yang et al. 2022; Kapelanski-Lamoureux et al. 2022). About 90% of patients with severe hemophilia aged over 35 years are infected with HCV (Dai et al. 1995). Additionally, co-infection with HCV and HIV exacerbates liver conditions in patients with hemophilia (Thalappillil et al. 2019). Although gene therapy is generally considered a safe treatment option for hemophiliacs, there has been speculation about its potential genotoxicity leading to the development of HCC. Studies have been conducted to assess the risk of carcinogenesis. Specific gene therapy studies for hemophilia B using rAAV vectors (FIX) in mice, dogs, and nonhuman primates suggested integration of AVV vectors into the genome without evidence for insertional oncogenesis (George et al. 2020; Schmidt et al. 2023; Ferla et al. 2020). Research by Denise E. Sabatino and colleagues on newborn mice showed a sevenfold increase in HCC susceptibility in individuals who received the vector compared to controls (Sabatino et al. 2022). The vector integration site was mapped to the Rian locus. Introducing the vector with a strong Rian locus-specific promoter resulted in HCC development in all newborn mice. However, no evidence of HCC development was observed in healthy adult mice following AAV vector therapy. It was hypothesized that the high frequency of vector integration is caused by proliferating hepatocytes, which are highly active in developing livers of newborn mice compared to the resting activity of hepatocytes in healthy adults. In conditions of chronic inflammation and liver damage, such as non-alcoholic fatty liver disease (NAFLD) or obesity, compensatory proliferation increases the risk of oncogenesis (Schmidt et al. 2023; Tsuchida et al. 2018). Research by Dhwanil A. Dalwadi on adult mice showed that liver proliferation and damage increased the risk of HCC in adults receiving rAAV vector therapy. The animals were infected with the wAAV C57BL/6 editing vector targeting the Rian locus, underwent partial hepatectomy to induce compensatory proliferation of hepatocytes, and were placed on a high-fat diet (HFD) to induce liver damage. It has been shown that hepatocyte proliferation caused by chronic liver damage and partial hepatectomy increases the risk of liver oncogenesis. The human ortholog of the mouse Rian locus is MEG8, making it challenging to speculate whether the human liver is equally susceptible to random integration of rAAV leading to the development of HCC. Animal models may be considered poorly predictive, and the integration of rAAV and the associated cancer risk in humans require further evidence from patient populations. Continued research is necessary to assess the risk of carcinogenesis, specifically HCC, in patients following the use of rAAV in gene therapy (Dalwadi et al. 2021).

During the initial systemic AAV gene therapy approved for treating hemophilia B, a case of HCC was reported in a patient who received etranacogene dezaparvovec. This therapy included the rAAV 5 vector carrying the Padua variant of the FIX transgene and a liver-selective promoter (Pipe et al. 2023; Thornburg 2021).

In the study led by Schmidt M. and colleagues, molecular analysis of the rAAV sequence in cells derived from a tumor lesion corresponding to HCC and adjacent cells revealed comparable integration of the vector (Schmidt et al. 2023). Integration events were dispersed across the genome. Numerous fusion sequences were detected in both samples, indicating the predominant episomal form of the vector genomes. If HCC development was linked to vector integration, one would expect frequent integration sites to predominate over the episomal form particularly near known HCC oncogenes such as *TP53* and *NFE2L2*. Furthermore, sequencing of the patient’s entire genome identified a deletion on chromosome 8, which is common in nearly 50% of HCC unrelated to gene therapy. Mutations in *TP53*, *NFE2L2*, and *PTPRK* were also detected. The patient had multiple HCC risk factors, including prior infections with hepatitis B and C viruses, advanced age, smoking history, and a family cancer history. In summary, molecular integration analysis of an HCC case following AAV gene therapy did not establish a direct link between rAAV5 vector administration and HCC occurrence. Currently, the risk of oncogenesis from rAAV therapy in humans remains theoretical (Schmidt et al. 2023).

## IX-R338L mutation (Padua)

Detection a naturally occurring gain-of-function (Padua) mutation in humans was a significant breakthrough in gene therapy for hemophilia B. The IX-R338L mutation, characterized by replacing arginine with leucine at position 338 in the catalytic domain of factor IX, results in increased activity (Nathwani 2022). The heightened specific activity of factor IX-R338L stems from its increased interaction with its cofactor, factor VIIIa. Activated factor IX (IXa) forms an enzyme complex with active factor VIII (VIIIa) crucial for the proteolytic activation of factor X and the intrinsic coagulation pathway (Samelson-Jones et al. 2019). Preclinical studies in animals demonstrate comparable immunogenicity and thrombogenicity between FIX-R338L and wild-type FIX (FIX-WT) (Crudele et al. 2015). Furthermore, no antibody formation resulting from cross-reactivity with factor IX-WT was observed (Finn et al. 2012). Recombinant IX-R338L exhibits 8 to 12 times greater specific activity than unmutated FIX-WT (George et al. 2017; Simioni et al. 2009). This insight has proven pivotal in the efficacy of AAV-mediated gene therapy for hemophilia. Low vector doses were employed to induce therapeutic factor IX expression in hemophilia B patients, minimizing risks of cellular immune responses against capsid proteins (George et al. 2017). AAV gene therapy using the Padua variant enabled a fourfold reduction in therapeutic vector dose while achieving a sixfold increase in factor IX activity levels compared to studies using factor IX-WT (Samelson-Jones et al. 2019).

## Inhibitors post-gene therapy

The efficacy of gene therapy for hemophilia B hinges significantly on managing adverse immune responses to transgene products, genetically modified cells, and/or vectors. Various immunological reactions to the transgene are plausible, with inhibitor development, particularly neutralizing antibodies, being the most concerning. According to animal model studies, the risk of developing inhibitors following gene therapy is generally deemed low (George 2021).

In preclinical studies by Crudele et al. that used hyperfunctional factor IX (FIX-Padua, arginine 338 to leucine) in canine models of hemophilia B, expression of FIX-R338L post-gene transfer did not lead to inhibitor formation. Researchers observed that hepatic transgene expression resulted in the clearance of neutralizing antibodies (FIX inhibitors) and induction of immunological tolerance (Crudele et al. 2015; Finn et al. 2012). Similarly, murine models have demonstrated that liver-targeted gene therapy can induce immune tolerance to human FIX, effectively eliminating pre-existing high-titer inhibitors. Both phenomena are dependent on Treg activity and a reduction in plasma and memory B cells specific to FIX (Arruda and Samelson-Jones 2016). Long-term follow-up of four out of seven patients with severe hemophilia B treated with AAV2-hFIX16 revealed that none of the patients enrolled in a 12–15-year follow-up protocol showed evidence of FIX inhibitors at any point. However, persistent high titers of antibodies against various AAV serotypes were noted even 15 years after the infusion (George et al. 2020).

What is worth mentioning when considering the development of inhibitors post gene therapy, is that clinical trials exclude participants with current presence or past history of inhibitors, emphasizing the inclusion criteria focused on minimizing the risk of inhibitor development (George 2021; Arruda and Samelson-Jones 2016; Soroka et al. 2023; Krumb et al. 2021).

## Future plans for gene therapies

The genetic revolution in hemophilia treatment extends beyond viral vector-based gene therapy, encompassing innovative approaches designed to bypass the need for coagulation factors and overcome inhibitor challenges. Among these novel treatments are fitusiran, concizumab, and marstacimab. Fitusiran is a synthetic interfering RNA (siRNA) that, by combining with a Ga1NAc ligand, targets antithrombin (AT) messenger RNA (mRNA). The mechanism of action of the drug is the cleavage of AT mRNA, which inhibits the translation process and protein synthesis (Hu et al. 2019). AT is an anticoagulant enzyme that, after activation with heparin, neutralizes, among others, factor X and thrombin. Indirectly, fitusiran allows to achieve higher thrombin values, which can restore homeostatic balance in patients with hemophilia B. Fitusiran therapy can be compared to balancing between a state of hypercoagulability (decreased level of AT and impaired blood coagulation (reduced level of factor IX in hemophilia B), which, if the right proportions are achieved, may become a universal solution for many patients. Research on its use is currently in phase 3 and gives promising results (Young et al. 2023a, b; Boyce and Rangarajan 2023; Pasi et al. 2021; Kenet et al. 2024). Additionally, siRNA therapies targeting protein S and heparin II cofactor are under preclinical investigation (Prince et al. 2020; Lin et al. 2021).

Concizumab is an IgG4 monoclonal antibody and inhibits tissue factor pathway inhibitor (TFPI) by binding to its Kunitz-2 domain. This prevents TFPI from binding to active factor X, thereby preventing factor X inhibition. The effect of the antibody is to increase thrombin production and thus reduce the number of bleeding events. As in the case of fitusiran, the use of concizumab requires special attention due to the increased level of prothrombotic factor and the risk of possible thromboembolic events. Concizumab is currently used in Canada, Switzerland, and Australia, among others (Matsushita et al. 2023; Keam 2023; Pasca 2022). Marstacimab, another TFPI undergoing clinical trials, the IgG1 monoclonal antibody and also the Kunitz-2 domain of the TFPI, presenting a similar mechanism of action (Donley et al. 2023).

An important consideration regarding AVV-based gene therapy concerns how long FIX expression will be possible after the introduction of the transgene. Vector therapy involves delivering the correct mRNA to liver cells, which does not integrate with the host genome but exists in the form of an episome. Due to hepatocyte division, the amount of introduced genetic material in these cells may gradually decrease. Therefore, there are concerns that AAV vector therapy may not be sustainable, especially in pediatric patients whose hepatocytes proliferate rapidly, resulting by using integrating vectors, such as lentiviral vectors, although their use a higher risk of mutagenesis (Milani et al. 2022).

An alternative approach could be gene editing method tested in animal models. The CRISPR/Cas system stands out as the most widely adopted strategy for this purpose. Other investigated methods include, among others, transcriptor activator-like effector nucleases (TALEN) and zinc-finger nucleases (ZFN) (Maeder and Gersbach 2016; Sung et al. 2019). The undeniable advantage of the CRISP/Cas strategy over other gene editing techniques is its simplicity and cost-effectiveness (Janik et al. 2020). CRISP/Cas consists of Cas genes encoding nucleases and short palindromic repeats (CRISPR), which are located between viral sequences integrated into the DNA (Hu and Li 2022; Pablo-Moreno et al. 2023). Animal tests focus on correcting induced pluripotent stem cells (iPSCs) isolated from the patient using CRISPR/Cas, differentiating them into hepatocytes or adult endothelial cells and subsequently introducing them into the body, often using the AAV vectors (Sidonio et al. 2021; De Wolf et al. 2023). Research in gene editing is continuously advancing with ongoing exploration of newer approaches.

## Conclusions

The long-awaited gene therapy for hemophilia B using viral vectors became a reality following approval by the European Medicine Agency (EMA) of etranacogene dezaparvovec therapy, now used in adults with moderate and severe forms of the disease. Despite initial concerns regarding potential side effects, the therapy has proven to be highly successful and could pave the way for alternative and innovative projects aimed at further enhancing the quality of life of patients.
